# Artery of Percheron Infarction: A Rare But Important Cause of Bilateral Thalamic Stroke

**DOI:** 10.7759/cureus.37054

**Published:** 2023-04-03

**Authors:** Tajah M Alaithan, Husam M Almaramhi, Afnan S Felemban, Abdullah M Alaithan, Ahlam Alharbi

**Affiliations:** 1 General Practice, Almaarefa University, Riyadh, SAU; 2 General Practice, University of Jeddah, Jeddah, SAU; 3 General Practice, Heraa General Hospital, Mecca, SAU; 4 General Practice, Al-Omran General Hospital, Al-Ahsa, SAU; 5 Family Medicine, Primary Health Care, Riyadh, SAU

**Keywords:** case report, hemiparesis, magnetic resonance imaging, computed tomography, bilateral thalamic, stroke, artery of percheron

## Abstract

Artery of Percheron infarction is a serious but rare condition that can result in acute bilateral thalamic infarction and a wide range of neurological symptoms. It occurs due to occlusion of the single arterial branch that supplies the medial thalamus and rostral midbrain bilaterally. In this case report, we describe a 58-year-old female with a history of hypertension and hyperlipidemia who presented with sudden confusion, speech difficulties, and right-sided weakness. An initial CT scan showed ill-defined hypodensity in the left internal capsule, which, when combined with the clinical features, suggested acute ischemic stroke. The patient received an IV tissue plasminogen activator within the recommended time window. Several days later, repeated imaging showed bilateral thalamic hypodensity consistent with subacute infarction in the territory of the artery of Percheron. The patient was subsequently discharged to a rehabilitation facility for further recovery and rehabilitation with residual mild hemiparesis. It is important for healthcare providers to maintain a high index of suspicion for the artery of Percheron infarction and be aware of its potential to cause acute bilateral thalamic infarction and a variety of neurological symptoms.

## Introduction

Artery of Percheron infarction is a rare but potentially devastating cause of acute bilateral thalamic infarction. The artery of Percheron is a single arterial branch that arises from the proximal basilar artery and supplies the medial thalamus and rostral midbrain bilaterally [1]. Occlusion of this artery can lead to acute bilateral thalamic infarction, which can result in a wide range of neurological symptoms and deficits. The first case of artery of Percheron infarction was reported in 1973 by French neurologist Gerard Percheron, who described a patient with a unique pattern of bilateral thalamic infarction [2-3]. Since then, only a few hundred cases have been reported in the literature, making this a rare but important clinical entity. The incidence of artery of Percheron infarction is estimated to be less than 1% of all strokes [1]. Early recognition and prompt management can improve outcomes.

## Case presentation

We present the case of a 58-year-old female who presented to the emergency department with sudden onset confusion, inability to speak, and right-sided weakness lasting one hour. The patient had a history of hypertension and hyperlipidemia and was not taking any medications at the time of presentation. On examination, she was disoriented with a Glasgow Coma Scale score of 12/15. She had right-sided hemiparesis with a National Institutes of Health Stroke Scale score of 12, impaired vertical gaze, and mild dysarthria. The patient's blood pressure was 180/100 mmHg, and her heart rate was 90 beats per minute.

**Figure 1:**
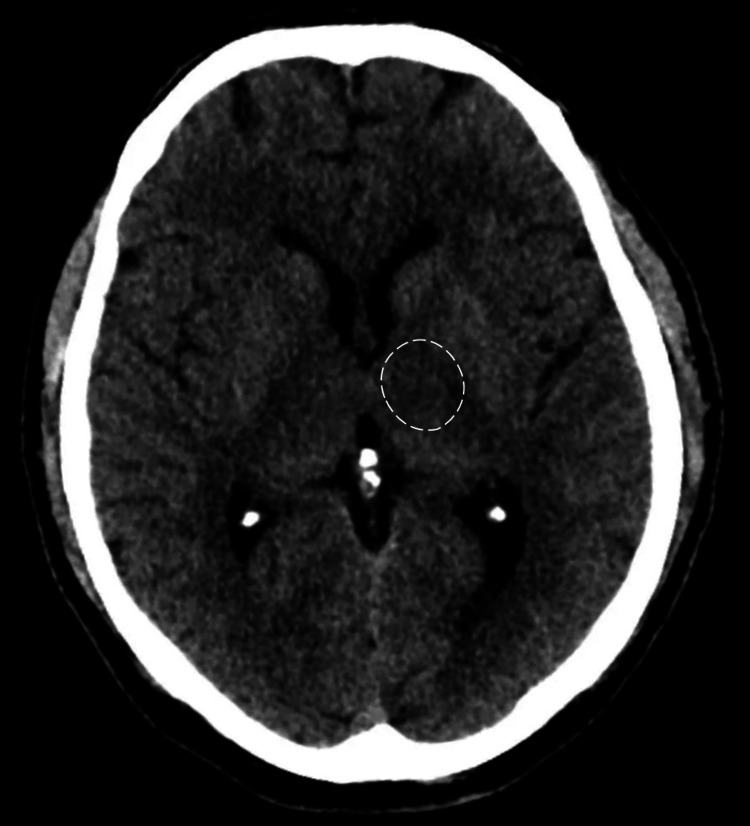
Axial CT scan of the head demonstrating ill-defined hypodensity in the posterior limb of the left internal capsule (arrow), indicative of acute ischemic stroke, in correlation with clinical findings of right hemiparesis.

The initial non-contrast head CT showed ill-defined hypodensity in the posterior limb of the left internal capsule, that is, in conjunction with the clinical finding of right hemiparesis which is suggestive of acute ischemic infarction (Figure 1). The patient was started on a statin and antihypertensive medication due to some blood pressure readings above 180/105 mmHg. She then received an IV tissue plasminogen activator within the recommended time window and was transferred to the neurology intensive care unit (ICU) for close monitoring and further management. IV antihypertensive medications were administered to control her blood pressure and prevent further brain injury, with her blood pressure maintained between 120/80 and 140/90 mmHg throughout her hospital stay. Aspirin was administered to the patient 72 h after thrombolysis. The patient also underwent a complete cardiac workup, which was unremarkable. Basic laboratory investigations showed no significant abnormalities.

**Figure 2:**
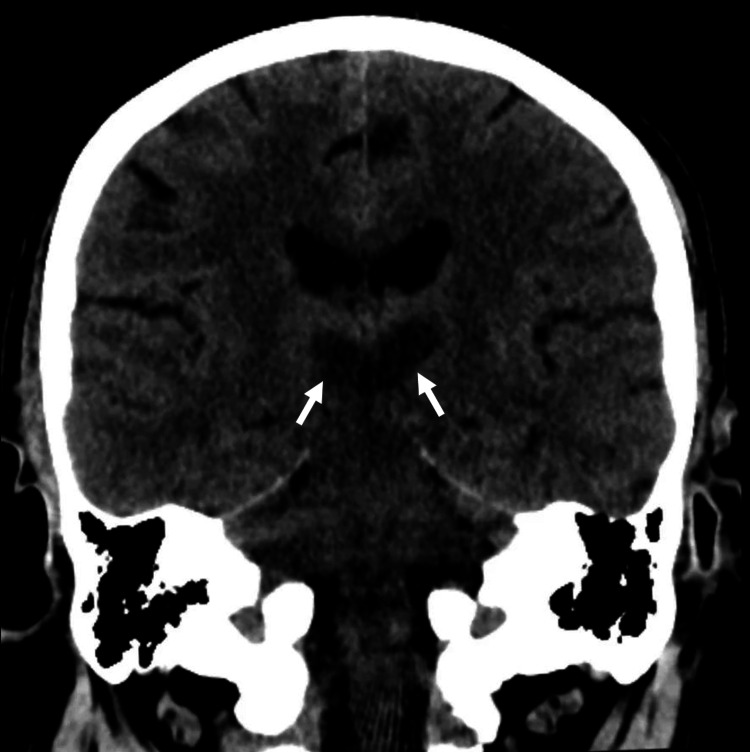
Coronal CT head image demonstrates bilateral thalamic hypodensity (arrows), which is highly suggestive of artery of Percheron infarction.

**Figure 3:**
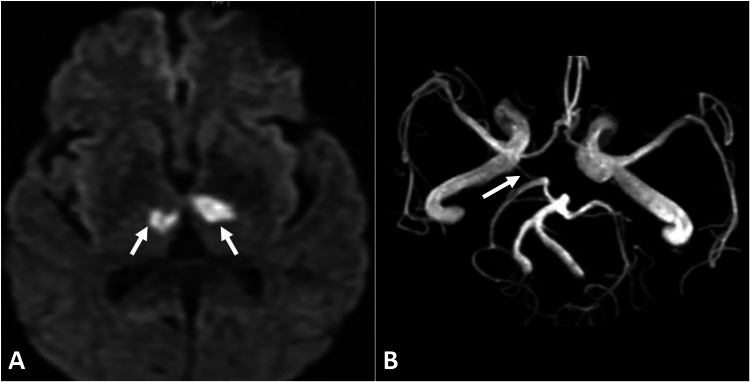
Diffusion-weighted imaging (A) reveals restricted diffusion in the bilateral thalami (arrows), and MR angiography (B) shows a single arterial trunk (arrow) supplying the thalami, which is consistent with the artery of Percheron infarction. MR: magnetic resonance

Three days later, a repeated CT scan showed hypodensity in the bilateral thalami consistent with acute infarction in the territory of the artery of Percheron (Figure 2). MRI confirmed the diagnosis, demonstrating restricted diffusion in the bilateral thalami and the rostral midbrain and revealing a single arterial trunk supplying the bilateral paramedian thalami (Figure 3).

Over the next few days, the patient showed gradual improvement in her symptoms. Her Glasgow Coma Scale score improved to 14/15, and her National Institutes of Health Stroke Scale score decreased to 8. The patient's neurological examination showed improvement, with partial recovery of right-sided strength and dysarthria. The patient developed mild dysphagia during her hospital stay, which was managed conservatively with a modified diet and speech therapy.

After a week of hospitalization, the patient was discharged to a rehabilitation facility for further recovery and rehabilitation. She continued to improve during her rehabilitation and was eventually discharged home with residual mild hemiparesis. The patient had a Modified Rankin Scale (mRS) score of 2, indicating slight disability, but was able to look after her own affairs without assistance although unable to carry out all previous activities. The Medical Research Council (MRC) muscle power score was 4/5. The patient was counseled on lifestyle modifications and the importance of adhering to medications for blood pressure and lipid control.

## Discussion

Artery of Percheron infarction is a rare but potentially devastating cause of acute bilateral thalamic infarction. Due to its rarity, diagnosis and management can be challenging, and the clinical course can vary widely depending on the extent and location of the infarction [2]. Our case report highlights the importance of early recognition and prompt management of this condition to improve outcomes.

The clinical presentation of the artery of Percheron infarction can be diverse and often depends on the extent of the infarction [3]. The most common symptoms include altered mental status, behavioral changes, and vertical gaze palsy, which were observed in our patient. These symptoms can be nonspecific and may mimic other neurological conditions, making the diagnosis challenging [2-3].

The diagnosis of artery of Percheron infarction can be challenging, and a high index of suspicion is required. Non-contrast head CT is usually the first imaging modality used in the emergency department [4]. However, MRI with diffusion-weighted imaging is more sensitive and specific in detecting acute infarction in the thalamus and midbrain [3]. It is also important to rule out other potential causes of bilateral thalamic infarction, such as metabolic disorders, venous infarction, vasculitis, and neoplasms, which were not present in our patient [5-6].

The prognosis of the artery of Percheron infarction varies depending on the extent and location of the infarction. Patients with extensive infarctions have a higher mortality rate and are more likely to have severe neurological deficits [3-4]. However, some patients may recover with minimal deficits, especially with early diagnosis and prompt management. It is important to note that patients with the artery of Percheron infarction may have residual deficits, such as hemiparesis, dysarthria, and cognitive impairment, which can affect their quality of life [6].

## Conclusions

Artery of Percheron infarction is a rare but significant cause of acute bilateral thalamic infarction. Despite its rarity, it is important for healthcare providers to be aware of this clinical entity as early recognition and prompt management can improve patient outcomes. The diagnosis of artery of Percheron infarction can be challenging, and a high index of suspicion is required. Imaging modalities, such as MRI with diffusion-weighted imaging and magnetic resonance angiography, can aid in the diagnosis of this condition.
